# Prevalence and associated family and lifestyle factors of precocious puberty in children: A retrospective observational study

**DOI:** 10.1097/MD.0000000000050764

**Published:** 2026-09-18

**Authors:** Kai Chen, Youli Li, Huasheng Jin

**Affiliations:** a Pediatric Department, Ningbo Yinzhou No.2 Hospital, Ningbo, Zhejiang Province, China.

**Keywords:** associated factors, children, family environment, lifestyle habits, precocious puberty, prevalence

## Abstract

Precocious puberty is an endocrine-related developmental disorder characterized by the premature onset of secondary sexual characteristics. Early pubertal development may adversely affect physical growth, psychosocial well-being, and long-term health. This study investigated the hospital-based proportion of children diagnosed with precocious puberty and explored associated family and lifestyle factors. We conducted a single-center retrospective observational study with an age- and sex-matched case-control analytic component, using clinical and questionnaire data collected from pediatric patients treated at our hospital between January 2021 and January 2023. Among 6021 screened children, 53 diagnosed with precocious puberty were included as cases, and 53 age- and sex-matched children with normal growth and development served as controls. Demographic characteristics, family background, and lifestyle habits were compared between groups. Exploratory correlation analysis was used to assess associations between selected family/lifestyle variables and precocious puberty status. The proportion of children diagnosed with precocious puberty among the 6021 surveyed pediatric patients was 0.88% (53/6021). No significant between-group differences were found in baseline demographic variables. However, children with precocious puberty more often had parents with unstable marital status, mothers with lower educational attainment, maternal employment outside the home, and shorter parental companionship time. In addition, late bedtime, reduced weekly exercise, and preference for emotionally oriented screen-based entertainment were more common in the precocious puberty group. Exploratory correlation analysis was consistent with associations between these family and lifestyle factors and precocious puberty status. In this single-center hospital-based cohort, 0.88% of surveyed pediatric patients were diagnosed with precocious puberty. Precocious puberty status was associated with several family- and lifestyle-related characteristics, including parental marital status, maternal education and occupation, parental companionship time, bedtime, physical activity, and hobby patterns. Because this observational study did not include multivariable adjustment, these findings should be interpreted as associations rather than independent or causal effects. Prospective studies with multivariable analyses are needed to determine whether these factors have independent or modifiable relationships with pubertal timing.

## 1. Introduction

Precocious puberty is characterized by the appearance of secondary sexual characteristics before 8 years of age in girls and before 9 years of age in boys.^[1–4]^ In girls, breast development (thelarche; Tanner breast stage B2) is the principal clinical sign of pubertal onset, whereas in boys, testicular enlargement to a volume of at least 4 mL is the principal sign.^[4]^ Accelerated linear growth and advancement of bone age may provide supportive evidence of progressive pubertal activation but are not, by themselves, equivalent to the age-based definition of precocious puberty. Domestic studies have shown that, with economic development and changes in living conditions, the timing of pubertal development has shifted earlier in some populations. Early pubertal development may affect final height potential and psychosocial well-being and has therefore attracted increasing clinical attention.^[4,5]^ Precocious puberty is multifactorial, with potential contributions from organic disease, genetic susceptibility, nutrition, family environment, living conditions, and socioeconomic factors.^[6–8]^ Accordingly, this study used hospital-based clinical and questionnaire data to estimate the proportion of surveyed pediatric patients diagnosed with precocious puberty and to examine family and lifestyle characteristics associated with precocious puberty status.

## 2. Materials and methods

### 2.1. Ethical approval

This study was approved by the Ethics Committee of Ningbo Yinzhou No.2 Hospital in accordance with regulatory and ethical guidelines pertaining to retrospective research studies. All procedures followed were in accordance with the ethical standards of the responsible committee on human experimentation and with the Helsinki Declaration. Written informed consent was obtained from the parents or legal guardians of all participating children prior to enrollment.

### 2.2. Study subjects

From January 2021 to January 2023, we retrospectively analyzed clinical and questionnaire data from 6021 pediatric patients evaluated at our hospital. The study therefore represents a single-center retrospective observational study; for the analytic comparison of associated factors, all 53 children who met the diagnostic criteria for precocious puberty were included as cases and were compared with 53 age- and sex-matched controls with normal growth and development from outpatient visits during the same period. Control participants were confirmed by clinical examination to have no evidence of early pubertal signs, endocrine disorders, or chronic systemic diseases. Because the questionnaire and clinical information had been collected during routine visits before the retrospective analysis, the study did not assign exposures or interventions. Data collection occurred between 2021 and 2023, overlapping with the coronavirus disease 2019 pandemic; pandemic-related changes in family interaction, screen exposure, sleep, and physical activity therefore remain a potential source of residual bias and are considered in the limitations.

### 2.3. Inclusion and exclusion criteria

Children were eligible for inclusion in the precocious puberty group if they met all of the following criteria: onset of secondary sexual characteristics before 8 years of age in girls or before 9 years of age in boys. In girls, pubertal onset was identified primarily by breast development (thelarche; Tanner breast stage B2) before 8 years; menarche, when present, was recorded as a later pubertal milestone rather than used as an alternative age threshold for diagnosis. In boys, pubertal onset was identified primarily by testicular enlargement to a volume of at least 4 mL and/or progressive genital development before 9 years. Clinical evidence of progressive pubertal development, assessed by Tanner staging and serial clinical information where available. Accelerated growth velocity and/or bone-age advancement beyond chronological age were considered supportive findings of progressive hypothalamic–pituitary–gonadal axis activation rather than stand-alone diagnostic criteria. Age ≤12 years at diagnosis. Newly diagnosed cases presenting for the first time to our hospital during the study period. The control group consisted of age- and sex-matched children with normal physical development, no signs of precocious puberty, and no underlying endocrine or systemic disorders.

Children were excluded from the study group if they met any of the following conditions: precocious puberty attributable to secondary causes, such as gonadal or adrenal tumors, congenital adrenal hyperplasia, or other identified organic etiologies; presence of severe dysfunction or failure of major organs (e.g., heart, liver, kidney), central nervous system malformations, or intracranial tumors; history of cerebral palsy, chromosomal abnormalities, or other endocrine/metabolic disorders known to affect growth and pubertal development; radiological evidence of hypothalamic–pituitary organic lesions on imaging studies; documented psychiatric illness, impaired consciousness, or communication difficulties that precluded reliable assessment; incomplete clinical or questionnaire data preventing inclusion in the final analysis.

### 2.4. Process

Assessment of sexual development: breast and external genital development were assessed using Tanner staging and standard charts.^[9]^ For girls, breast Tanner stage B2 before 8 years was considered the principal clinical sign of precocious pubertal onset; for boys, testicular enlargement to at least 4 mL and/or progressive genital development before 9 years was used as the principal clinical sign. Information on menarche and nocturnal emissions was obtained through questionnaires and parent interviews as supplementary pubertal history. Growth velocity and bone age were used as supportive indicators of pubertal progression. The same standardized questionnaire, item wording, interviewer guidance, and face-to-face administration procedure were used in both groups. For the precocious puberty group, the questionnaire was administered at the time of diagnosis, immediately after confirmation by pediatric endocrinologists; for controls, it was administered during outpatient visits in the same study period after confirmation of normal development.

The questionnaire used in this study was adapted from a previously published master’s thesis on precocious puberty in Chinese children,^[10]^ with reference to established literature on potentially relevant family and lifestyle factors. Pediatric experts with the title of associate chief physician or above reviewed the instrument and refined the items for clinical relevance, comprehensibility, and content coverage, providing a qualitative assessment of face/content validity. All investigators received standardized training in administration procedures, interviewing techniques, and questionnaire coding. Before formal data collection, a pilot investigation was conducted to assess feasibility, item clarity, respondent comprehension, and the administration process, and wording or procedural problems identified during the pilot were corrected. No quantitative psychometric assessment (such as Cronbach’s alpha, test–retest reliability, or a numerical content–validity index) was performed or available for the modified questionnaire. After parents or legal guardians had been informed and provided consent, questionnaires were administered face-to-face in the hospital using the same standardized procedure for cases and controls.

### 2.5. Survey indicators

The diagnosis of precocious puberty was made independently by 2 experienced pediatric endocrinologists based on clinical history, physical examination, and Tanner staging criteria. The incidence rate of precocious puberty was then calculated among the surveyed pediatric patients.

The questionnaire used in this study was adapted from an instrument developed in a previous thesis study on Chinese children,^[10]^ with modifications reviewed by pediatric experts for face/content validity. The survey included the following domains: general information: child’s sex, age, body mass index (BMI), living environment (urban/rural), only-child status, and mode of delivery (vaginal/cesarean). Family factors: parental marital status, maternal education level, maternal occupation, and weekly time parents spent with the child. Lifestyle factors: bedtime, presence of afternoon nap habit, weekly exercise time, and preferred hobbies (watching television dramas, reading, gaming, or other activities). The identical questionnaire and administration protocol were used for cases and controls. All questionnaires were completed face-to-face by parents under the guidance of trained investigators, and responses were checked for completeness and consistency before data entry.

### 2.6. Statistical methods

All statistical analyses were performed using IBM Statistical Package for the Social Sciences Statistics version 26.0 (IBM Corp.). Categorical variables were expressed as frequencies and percentages, and comparisons between groups were conducted using the chi-square test or Fisher exact test, as appropriate. Continuous variables were assessed for normality; approximately normally distributed variables were presented as mean ± standard deviation and compared using the independent-samples *t* test. For the exploratory correlation analysis, precocious puberty status was coded as 0 = control and 1 = precocious puberty. Dichotomous family variables were coded so that the category hypothesized to reflect greater social or lifestyle adversity received the higher value (parental marital status: first marriage = 0, unmarried/divorced/remarried = 1; maternal education: above junior high school = 0, junior high school or below = 1; maternal occupation: fixed job = 0, migrant/flexible work outside the home = 1). Parental companionship time and weekly exercise time were treated as ordered categories, with higher scores representing less companionship and less exercise, respectively. Bedtime was coded as after 23:00 = 0 and before 23:00 = 1 so that the reported coefficient represented the association with earlier bedtime. For hobbies, responses were dichotomized for correlation analysis as preference for idol/romance-oriented television programs = 0 versus other hobbies = 1. Pearson correlation coefficients (*r*) were calculated using these prespecified numeric codes; for binary variables this is equivalent to a point-biserial/phi-type correlation. *R*^2^ values, where reported, were the squared Pearson correlation coefficients and were used only as descriptive measures of shared variation, not as estimates of causal or independent effects. The correlation analysis was exploratory, and no multivariable adjustment was performed. All statistical tests were two-sided, and *P* < .05 was considered statistically significant.

## 3. Results

### 3.1. General information

As shown in Table 1, there were no statistically significant differences in baseline demographic and perinatal characteristics between the precocious puberty group and the healthy control group (all *P* > .05). Specifically, the distribution of sex was comparable between the 2 groups (*P* = .845). The mean age did not differ significantly (*P* = .118). Similarly, BMI showed no significant difference (*P *= .197). In addition, there were no significant differences in living environment (urban vs rural, *P* = .841), only-child status (*P* = .821), or mode of delivery (cesarean vs vaginal, *P* = .647). These findings indicate that the 2 groups were generally comparable in their basic demographic and perinatal profiles.

**Table 1:** Comparison of general information.

	Healthy group (n = 53)	Precocious puberty group (n = 53)	*t*	*P* value
Gender (male/female)	31 (58.49%)/22 (41.51%)	29 (54.72%)/24 (45.28%)	0.038	.845
Age	6.52 ± 1.24	6.21 ± 0.75	1.580	.118
BMI (kg/m^2^)	16.86 ± 1.92	17.38 ± 2.14	1.299	.197
Living environment (rural/urban)	21 (39.62%)/32 (60.38%)	19 (35.85%)/34 (64.15%)	0.040	.841
Only child (yes/no)	39 (73.58%)/14 (26.42%)	41 (77.36%)/12 (22.64%)	0.051	.821
Mode of delivery (vaginal/cesarean section)	39 (73.58%)/14 (26.42%)	42 (79.25%)/11 (20.75%)	0.209	.647

BMI = body mass index.

### 3.2. Family status

As shown in Table 2, significant differences were observed between the 2 groups in parental marital status, maternal education level, maternal occupation, and parental companionship time (all *P* < .05). In terms of marital status, the proportion of children from unmarried, divorced, or remarried families was higher in the precocious puberty group than in the healthy group (43.40% vs 9.43%), whereas the proportion of children with parents in their first marriage was lower (56.60% vs 90.57%, *P* < .001). With respect to maternal education, 56.60% of mothers in the precocious puberty group had an education level of junior high school or below, compared with 33.96% in the healthy group, while a higher proportion of mothers in the control group had attained education above junior high school (66.04% vs 43.40%, *P* = .032). Maternal occupation also differed significantly: in the precocious puberty group, 60.38% of mothers were engaged in migrant or flexible work outside the home, whereas 67.92% of mothers in the healthy group held fixed jobs (*P* = .006). Parental companionship time was lower in the precocious puberty group: 79.25% of parents spent less than 2 hours per week with their children, compared with 18.87% in the healthy group. In contrast, 47.17% of the healthy group reported more than 5 hours of weekly companionship, versus 11.32% in the precocious puberty group (*P* < .001). These findings show unadjusted associations between precocious puberty status and parental marital status, maternal education and occupation, and parental companionship time; they do not establish that any 1 factor has an independent effect.

**Table 2:** Comparison of family status.

	Healthy group (n = 53)	Precocious puberty group (n = 53)	*t*	*P* value
Parental marital status				
Unmarried	1 (1.89%)	4 (7.55%)	None	<.001
First marriage	48 (90.57%)	30 (56.60%)		
Divorced	2 (3.77%)	12 (22.64%)		
Remarried	2 (3.77%)	7 (13.21%)		
Mother’s education				
Junior high school or below	18 (33.96%)	30 (56.60%)	4.607	.032
Junior high school or above	35 (66.04%)	23 (43.40%)		
Mother’s occupation				
Working outside the home	17 (32.08%)	32 (60.38%)	7.439	.006
Fixed job	36 (67.92%)	21 (39.62%)		
Parental companionship				
5 h/wk	25 (47.17%)	6 (11.32%)	38.685	<.001
2–5 h/wk	18 (33.96%)	5 (9.43%)		
<2 h/wk	10 (18.87%)	42 (79.25%)		

### 3.3. Children’s lifestyle habits

As summarized in Table 3, significant differences were observed between the 2 groups in bedtime, exercise time, and hobbies, whereas nap habits showed no significant variation. With regard to bedtime, most children in the healthy group went to bed before 23:00 (75.47%), while over half of those in the precocious puberty group went to bed after 23:00 (52.83%, *P* = .005). Afternoon nap habits did not differ significantly between groups (*P* = .120). Physical activity levels also differed: in the healthy group, 50.94% of children exercised more than 7 hours per week, whereas in the precocious puberty group, 60.38% reported less than 1 hour of weekly exercise (*P* < .001). Regarding hobbies, 73.58% of children in the healthy group reported diverse interests beyond screen-based entertainment, compared with 5.66% in the precocious puberty group. In contrast, 60.38% of the precocious puberty group reported a preference for idol dramas, compared with 5.66% of the healthy group (*P* < .001). These findings indicate unadjusted associations of precocious puberty status with later bedtime, lower reported physical activity, and hobby pattern.

**Table 3:** Comparison of children’s lifestyle habits.

	Healthy group (n = 53)	Precocious puberty group (n = 53)	*t*	*P* value
Bedtime				
Before 23:00	40 (75.47%)	25 (47.17%)	7.796	.005
After 23:00	13 (24.53%)	28 (52.83%)		
Afternoon nap habit				
Yes	31 (58.49%)	22 (41.51%)	2.415	.120
No	22 (41.51%)	31 (58.49%)		
Exercise time				
More than 7 h/wk	27 (50.94%)	6 (11.32%)	34.066	<.001
1–7 h/wk	21 (39.62%)	15 (28.30%)		
Less than 1 h/wk	5 (9.43%)	32 (60.38%)		
Hobbies				
Idol dramas	3 (5.66%)	32 (60.38%)	65.158	<.001
Romance novels	3 (5.66%)	15 (28.30%)		
Gaming	8 (15.09%)	3 (5.66%)		
Other	39 (73.58%)	3 (5.66%)		

### 3.4. Correlation analysis

As presented in Table 4, the exploratory correlation analysis also identified associations between the prespecified coded family/lifestyle variables and precocious puberty status. Lower maternal education (*r* = 0.227, *P* = .019), maternal engagement in migrant or flexible work (*r* = 0.284, *P* = .003), and non-first-marriage parental status (*r* = 0.238, *P* = .014) were positively correlated with precocious puberty status under the coding described in the Materials and methods. Less weekly parental companionship showed a larger correlation (*r* = 0.558, *P* < .001). Earlier bedtime, coded as 1 versus 0 for bedtime after 23:00, was inversely correlated with precocious puberty status (*r* = −0.291, *P* = .003). Less weekly exercise time was positively correlated with precocious puberty status (*r* = 0.558, *P* < .001). For hobbies, the dichotomized variable (other hobbies = 1 vs idol/romance-oriented programs = 0) was inversely correlated with precocious puberty status (*r* = −0.767, *P* < .001), consistent with a higher frequency of idol/romance-oriented programs among cases. Because these are unadjusted correlations derived from coded categorical/ordinal variables, the coefficients should be interpreted as descriptive associations rather than independent effects or causal relationships.

**Table 4:** Correlation analysis of risk factors for precocious puberty.

Factors	*r*	*R* ^2^	*P* value
Parental marital status	0.238	0.057	.014
Mother’s education	0.227	0.052	.019
Mother’s occupation	0.284	0.081	.003
Parental time spent	0.558	0.311	<.001
Bedtime	−0.291	0.084	.003
Exercise time	0.558	0.311	<.001
Hobby	−0.767	0.589	<.001

## 4. Discussion

Precocious puberty is a common endocrine disorder in children, and its onset is very secretive and easily overlooked, often leading to delayed diagnosis and significant impacts on their psychological and behavioral development, which has attracted widespread attention from society.^[11,12]^ Because children’s intellectual and cognitive maturity lags behind their premature sexual development, early pubertal onset may provoke social difficulties and psychological distress.^[13,14]^ With the improvement of living conditions, changes in diet and lifestyle have contributed to an increasing incidence of precocious puberty worldwide.^[15]^ Previous studies have shown that the incidence of precocious puberty is around 0.12% to 0.19% in foreign countries,^[16]^ while domestic studies have reported an incidence of 0.43% to 4.74%.^[17]^ By providing hospital-based prevalence data (0.88%) in a Chinese cohort, our study adds to the limited regional epidemiological evidence, thereby helping to bridge the gap between national surveillance estimates and localized clinical observations.

The etiology of precocious puberty is multifactorial, involving genetic, nutritional, and environmental determinants.^[18,19]^ The present study showed no statistically significant differences in general information between the 2 groups (*P* > .05), but the BMI of children in the precocious puberty group was higher than that of the healthy group, which is consistent with previous research results,^[20]^ suggesting that the development of secondary sexual characteristics has a certain impact on children’s growth and development. However, further research is needed to determine the cause-and-effect relationship between these developmental indicators and childhood precocious puberty, and statistical analysis of family conditions and lifestyle habits is still needed to clarify the relationship between these factors and childhood precocious puberty. It is noteworthy that, unlike most published cohorts where girls predominate,^[1]^ our study showed a relatively high proportion of boys with precocious puberty. This discrepancy is likely attributable to the small sample size and potential selection bias, as parents of boys may be more likely to seek medical attention when signs of puberty appear unusually early. Therefore, the sex distribution in our study may not accurately reflect the true epidemiology of idiopathic central precocious puberty, which remains more common in girls. Although likely influenced by sample size, our observation underscores the need for more attention to early pubertal signs in boys, a group that is often underrepresented in epidemiological studies of precocious puberty.

In terms of family factors, parental marital status, maternal education, maternal occupation, and parental companionship time were associated with precocious puberty status in the unadjusted analyses. Limited parental companionship (<2 h/wk) showed one of the larger exploratory correlations (*r* = 0.558), an aspect that has been less frequently quantified in previous studies. These findings are compatible with a possible relationship between the psychosocial family environment and pubertal timing, although the present data cannot determine mechanism or causality. Psychosocial stress has been discussed as 1 potential pathway linking family environment and pubertal development,^[21]^ but maternal education, employment, family structure, and companionship time are interrelated and may also reflect broader socioeconomic circumstances. Therefore, our results should be interpreted as hypothesis-generating associations rather than evidence that increasing parental companionship or changing family structure would prevent precocious puberty.

Lifestyle characteristics were also associated with precocious puberty status. A later bedtime and lower reported physical activity were more common among cases, broadly consistent with literature linking sleep timing and sedentary behavior with pubertal development.^[22]^ We also observed an association between preference for idol/romance-oriented media and precocious puberty status (*r* = −0.767 under the coding described in the Materials and methods). This finding may reflect differences in screen exposure, age-related interests, family supervision, psychosocial context, or other unmeasured factors; the present observational data do not establish emotionally stimulating media as an environmental trigger of precocious puberty.^[23]^ Similarly, our data do not demonstrate that an earlier bedtime, increased exercise, or changes in hobbies have a protective effect against precocious puberty. These behaviors may nevertheless be reasonable targets for general child health promotion, while their specific relationship with pubertal timing should be examined in prospective studies with objective exposure measures and multivariable adjustment.^[24]^

This study has several limitations. The use of parent- and child-reported questionnaires may introduce recall and reporting bias regarding lifestyle habits. The relatively small sample size reduces precision and limited our ability to perform multivariable regression to assess independent associations and potential collinearity. The questionnaire focused mainly on family structure, parental education and occupation, and selected lifestyle habits; potentially relevant factors such as dietary patterns, nutritional status, endocrine-disrupting exposures, and detailed socioeconomic indicators were not assessed. In addition, parental education, maternal employment, parental companionship, and children’s lifestyle behaviors are likely interrelated, so their individual contributions cannot be separated in the present unadjusted analysis. The modified questionnaire underwent expert review and pilot testing for feasibility and item clarity, but no quantitative psychometric evaluation (e.g., internal consistency, test–retest reliability, or numerical content–validity index) was performed; this may affect measurement reliability and should be addressed in future studies. Although the same questionnaire and standardized face-to-face administration procedure were used for both groups, differential recall after a diagnosis of precocious puberty remains possible. The higher BMI observed among children with precocious puberty does not permit determination of temporal direction. Finally, data collection overlapped with the coronavirus disease 2019 pandemic, when changes in screen exposure, sleep, physical activity, and psychological stress may have affected measured behaviors.^[25]^ Larger multicenter prospective studies with validated instruments, objective lifestyle measures, and comprehensive multivariable modeling are needed to confirm these associations.

## 5. Conclusion

In summary, in this single-center retrospective observational study, precocious puberty status was associated with parental marital status, maternal education, maternal occupation, parental companionship time, bedtime, weekly exercise time, and hobby pattern. These findings represent unadjusted associations and should not be interpreted as evidence that these variables are independent risk factors, protective factors, or causal determinants of precocious puberty. Because the sample was small and no multivariable analysis was performed, residual confounding and interrelationships among family and lifestyle variables cannot be excluded. Future prospective, multicenter studies using validated measurement instruments and multivariable approaches are needed to determine the independent and potentially modifiable factors related to pubertal timing.

## Author contributions

**Conceptualization:** Kai Chen, Youli Li, Huasheng Jin.

**Data curation:** Kai Chen, Youli Li, Huasheng Jin.

**Formal analysis:** Kai Chen, Youli Li, Huasheng Jin.

**Funding acquisition:** Huasheng Jin.

**Investigation:** Huasheng Jin.

**Writing – original draft:** Huasheng Jin.

**Writing – review & editing:** Huasheng Jin.
